# The Relationship between T1 Sagittal Angle and Sagittal Balance: A Retrospective Study of 119 Healthy Volunteers

**DOI:** 10.1371/journal.pone.0160957

**Published:** 2016-08-11

**Authors:** Mingyuan Yang, Changwei Yang, Haijian Ni, Yuechao Zhao, Ming Li

**Affiliations:** 1 Department of Orthopedics, Changhai Hospital, Second Military Medical University, Shanghai, People’s Republic of China; 2 Department of Orthopedic Oncology, Changzheng Hospital, Second Military Medical University, Shanghai, People’s Republic of China; Harvard Medical School/BIDMC, UNITED STATES

## Abstract

T1 sagittal angle has been reported to be used as a parameter for assessing sagittal balance and cervical lordosis. However, no study has been performed to explore the relationship between T1 sagittal angle and sagittal balance, and whether T1 sagittal angle could be used for osteotomy guidelines remains unknown. The aim of our study is to explore the relationship between T1 sagittal angle and sagittal balance, determine the predictors for T1 sagittal angle, and determine whether T1 sagittal angle could be used for osteotomy guidelines to restore sagittal balance. Medical records of healthy volunteers in our outpatient clinic from January 2014 to August 2015 were reviewed, and their standing full-spine lateral radiographs were evaluated. Demographic and radiological parameters were collected and analyzed, including age, gender, T1 sagittal angle, maxTK, maxLL, SS, PT, and PI. Correlation coefficients between T1 sagittal angle and other spinopelvic parameters were determined. In addition, multiple regression analysis was performed to establish predictive radiographic parameters for T1 sagittal angle as the primary contributors. A total of 119 healthy volunteers were recruited in our study with a mean age of 34.7 years. It was found that T1 sagittal angle was correlated with maxTK with very good significance (r = 0.697, *P*<0.001), maxLL with weak significance (r = 0.206, *P* = 0.024), SS with weak significance (r = 0.237, *P* = 0.009), PI with very weak significance (r = 0.189, *P* = 0.039), SVA with moderate significance (r = 0.445, *P*<0.001), TPA with weak significance (r = 0.207, *P* = 0.023), and T1SPI with weak significance (r = 0.309, *P* = 0.001). The result of multiple regression analysis showed that T1 sagittal angle could be predicted by using the following regression equation: T1 sagittal angle = 0.6 * maxTK—0.2 * maxLL + 8. In the healthy population, T1 sagittal angle could be considered as a useful parameter for sagittal balance; however, it could not be thoroughly replaced for SVA. maxTK was the primary contributor to T1 sagittal angle. According to this equation, we could restore sagittal balance by surgically changing thoracic kyphosis and lumbar lordosis, which could serve as a guideline for osteotomy.

## Introduction

Many studies [1–4] have demonstrated that sagittal balance rather than coronal balance is significantly correlated with health-related quality of life (HRQOL), especially in patients who received surgical treatment because sagittal imbalance after spinal surgery may be a primary contributor to pain and disability. Therefore, more attention is often paid to sagittal balance than to coronal balance during the pre- and post-operative deformity assessment, surgical plan-making and surgical procedure [5,6].

Sagittal vertical axis (SVA) refers to the distance between the center of the body of C7 and the posterior-superior edge of S1, and is commonly used as gold standard to evaluate sagittal balance during assessment of spinal sagittal plane deformities [7–10]. Although SVA is regarded as the gold standard of evaluating sagittal balance, it is likely to produce measurement errors because it neglects the position of the head and cervical spine [9,10] and fails to take into account the pelvic compensation [11]. In addition, it is greatly affected by the patient’s posture. All these demerits have urged spinal surgeons to search for better parameters to assess sagittal balance.

T1 sagittal angle, the angle between a horizontal line and the cranial end plate of T1, is a novel parameter for evaluating the whole sagittal balance with fewer measurement errors because it takes into account the head position. Therefore, it is better correlated with SVA and could be utilized where long films cannot be obtained [9]. But whether T1 sagittal angle could represent sagittal balance more accurately than other sagittal parameters including SVA, TPA (T1 pelvic angle) and T1SPI (T1 spinopelvic inclination) remains unclear. In addition, our team has found great impacts of lumbar lordosis (LL) and thoracic kyphosis (TK) on maintenance and prediction of sagittal balance, which are considered novel regional predictors for sagittal balance [12,13]. Therefore, we speculated that LL and TK may be important contributors to T1 sagittal angle and sagittal balance, and we could restore sagittal balance through T1 sagittal angle by changing TK and LL in the surgical procedure. The aim of the present study was to explore the relationship between T1 sagittal angle and sagittal balance, compare T1 sagittal angle with other sagittal parameters, and determine the predictors for T1 sagittal angle in normal populations, hoping that the results of the study could provide guidance for osteotomy by changing these primary contributors in correction surgery.

## Materials and Methods

### Patient population

A total of 119 healthy volunteers in our outpatient clinic from January 2014 to August 2015 who met the inclusion and exclusion criteria were retrospectively reviewed. The inclusion criteria were as follows: 1) younger than 60 years; 2) no history of spinal disorders and spine surgery; and 3) no history of lower back pain (at least 6 months before participation in this study) and radiological abnormalities. The exclusion criteria were as follows: 1) a definite diagnosis of lumbar spinal pathology and spinal deformities, including tumors or infections; and 2) hip, knee and ankle abnormalities. Subjects without sufficient radiographic parameters were also excluded from our study. This study was approved by the Institutional Review Board of Changhai Hospital, and all patients in our study provided written informed consent for the study.

### Data collection

Demographic data including gender and age were collected. Radiographic parameters of the whole spine were measured in a lateral position by two surgeons independently, including T1 sagittal angle (the angle between a horizontal line and the superior end plate of T1), maxTK (thoracic kyphosis calculated by the Cobb method), maxLL (lumbar lordosis calculated by the Cobb method), SS (the angle between the horizontal and the sacral plate), PT (the angle between the vertical and the line through the midpoint of the sacral plate to femoral heads axis), PI (angle subtended by a perpendicular from the upper endplate of S1 and a line connecting the center of the femoral head to the center of the upper endplate of S1), SVA (the horizontal offset from the posterosuperior corner of S1 to the vertebral body of C7), T1 pelvic angle (TPA, the angle between the line from the femoral head axis to the centroid of T1 and the line from the femoral head axis to the middle of the S1 endplate), and T1SPI (T1 spinopelvic inclination, the angle between the line from the femoral head axis to the centroid of T1 and the plummet line). Correlation coefficients between T1 sagittal angle and other sagittal parameters were determined, multiple regression analysis was conducted to find out the primary contributors to T1 sagittal angle, and further, adjusted multiple regression analysis was conducted using morphologic parameters (maxTK and maxLL) to establish predictive radiographic parameters and formula for T1 sagittal angle.

### Statistical analysis

Statistical analyses were performed using SPSS 17.0 statistics software (SPSS Inc., Chicago, IL). Descriptive statistics were listed in the form of mean and standard deviation. T1 sagittal angle and its correlation with radiographic parameters were analyzed by correlation coefficient test. Unadjusted multiple regression analysis was performed to detect primary contributors to T1 sagittal angle using parameters that were significantly correlated with T1 sagittal angle in the correlation coefficients analysis, and adjusted multiple regression analysis was conducted to find out the regression equation using morphologic parameters (maxTK and maxLL) to predict T1 sagittal angle. *P*<0.05 was selected as significant level.

## Results

A total of 119 healthy volunteers (M: 61; F: 58) were recruited in our study with the mean age of 34.7 years. The mean T1 sagittal angle, maxTK, maxLL, SS, PT, PI, SVA, TPA and T1SPI were 19.76°, 35.80°, 50.18°, 34.34°, 12.95°, 47.29°, 2.76mm, 8.28° and -4.67° (Table 1). In addition, no significant difference in demographic and radiological parameters was observed between males and females (all *P*>0.05) (Table 2).

**Table 1:** General characteristics and radiographic parameters of the healthy population included in this study.

Variable	Mean	Standard deviation	Minimum	Maximum
Age (years)	34.71	13.81	11	58
T1 sagittal angle (°)	19.76	5.79	5	33
maxTK (°)	35.80	8.54	20	71
maxLL (°)	50.18	9.71	24	88
SS (°)	34.34	6.96	17	57
PT (°)	12.95	6.42	-2	27
PI (°)	47.29	9.58	28	78
SVA (mm)	2.76	21.59	-58	49
TPA (°)	8.28	5.82	-6	25
T1SPI (°)	-4.67	3.45	-13	5

**Table 2:** General characteristics in different genders.

Variable	Male (Mean±Standard deviation)	Female (Mean±Standard deviation)	*P* value
Age (years)	33.77±12.88	35.71±14.77	0.447
T1 sagittal angle (°)	19.70±5.82	19.81±5.81	0.921
maxTK (°)	35.56±9.18	36.05±7.88	0.754
maxLL (°)	50.36±10.75	50.00±8.57	0.840
SS (°)	34.89±7.28	33.78±6.62	0.387
PT (°)	13.44±5.18	12.43±7.51	0.397
PI (°)	48.33±9.41	46.21±9.71	0.229
SVA (mm)	4.80±21.50	0.60±21.67	0.291
TPA (°)	8.98±4.90	7.53±6.61	0.179
T1SPI (°)	-4.46±3.11	-4.90±3.79	0.494

**Fig 1:**
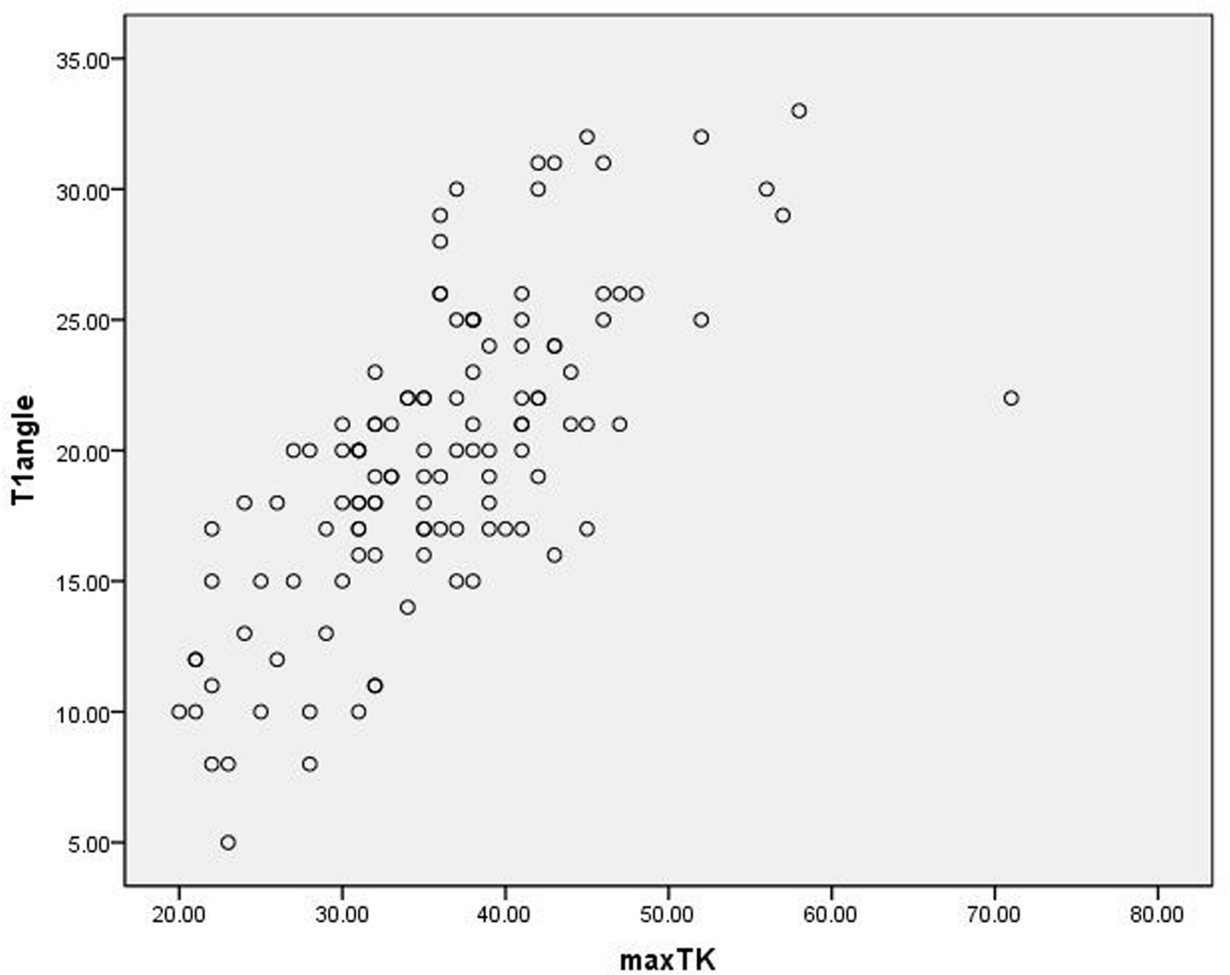
Correlation between T1 sagittal angle and maxTK for the healthy volunteers.

T1 sagittal angle was correlated with maxTK with very good significance (r = 0.697, *P*<0.001), maxLL with weak significance (r = 0.206, *P* = 0.024), SS with weak significance (r = 0.237, *P* = 0.009), PI with very weak significance (r = 0.189, *P* = 0.039), SVA with moderate significance (r = 0.445, *P*<0.001), TPA with weak significance (r = 0.207, *P* = 0.023), and T1SPI with weak significance (r = 0.309, *P* = 0.001), while no significant correlation was observed between T1 sagittal angle and the other radiological parameters (Table 3). In addition, a strong correlation was observed between T1 sagittal angle and maxTK (Fig 1).

**Table 3:** Correlation between T1 sagittal angle and other variables.

Variables	Pearson correlation coefficient (r)	*P* value
Age (years)	0.111	0.229
maxTK (°)	0.699	***P*<0.001**
maxLL (°)	0.209	**0.023**
SS (°)	0.239	**0.009**
PT (°)	0.023	0.806
PI (°)	0.189	**0.039**
SVA (mm)	0.452	***P*<0.001**
TPA (°)	0.208	**0.023**
T1SPI (°)	0.309	**0.001**

Unadjusted multiple linear regression analysis was conducted by using variables that were found to be significantly correlated with T1 sagittal angle in the correlation coefficients analysis, and the results suggested that maxTK, maxLL and SS were the primary contributors to T1 sagittal angle (all *P*<0.001), while there was no significant correlation between T1 sagittal angle and PI (*P* = 0.589) (Table 4). Since it was difficult to predict the postoperative values of compensatory parameters such as SS accurately before correction surgery; therefore, we removed SS from the multiple regression equation. Adjusted regression analysis showed that maxTK and maxLL were significantly associated with T1 sagittal angle, which could be predicted by using the following regression equation: T1 sagittal angle = 0.6 * maxTK—0.2 * maxLL + 8, as shown in Table 4.

**Table 4:** Multiple regression analysis on T1 sagittal angle and maxTK, maxLL, SS and PI.

	B	Standard error	t	*P* value
Unadjusted				
Constant	-0.067	0.764	-0.087	0.930
maxTK	0.982	0.021	45.730	***P*<0.001**
maxLL	-0.966	0.030	-32.163	***P*<0.001**
SS	0.951	0.041	22.997	***P*<0.001**
PI	0.010	0.019	0.542	0.589
Adjusted				
Constant	8.537	1.873	4.558	***P*<0.001**
maxTK	0.646	0.053	12.248	***P*<0.001**
maxLL	-0.237	0.046	-5.111	***P*<0.001**

## Discussion

Rebalancing sagittal balance is considered to be more important than correcting the coronal spinal deformity in the spinal surgery because sagittal balance has been demonstrated to be significantly correlated with HRQOL [14–17]. The whole sagittal balance can be evaluated by SVA, TPA, T1 sagittal angle and T9 sagittal offset [9,18,19]. However, each parameter has its own merits and demerits [9,11,18]. Among these parameters that reflect the whole sagittal balance, T1 sagittal angle might be an ideal measure compared with the others, and therefore could be used easily when long-standing radiographs are not available. in addition, T1 sagittal angle is associated with lesser measurement errors as compared with distance measurement such as SVA, although it also needs to be perfected.

Several methods and techniques have been used to restore sagittal balance during surgery in clinical practices [20–22]. Spine osteotomies, including Smith-Petersen osteotomy (SPO), pedicle subtraction osteotomy (PSO) and vertebral column resection (VCR) and etc. are the commonly used methods to restore alignment of the spine [23,24], although several disadvantages have been recognized, such as longer operation time, larger blood loss, and higher revision rates. Therefore, it is urgent to detect primary factors and predictors for sagittal balance so that we could restore sagittal alignment through changing these factors in the surgical process, which could provide guidance for osteotomy selection.

In our previous study, T1 sagittal angle, together with age and PT, was found to be one of the three primary contributors to maintaining sagittal balance. As pelvic parameters play a role in compensatory mechanisms [25], finding out the possible factors for T1 sagittal angle might be as equally important as finding out the predictors for the whole sagittal balance. In addition, our previous study [12,13] also found that LL and TK were important contributors to sagittal balance. Therefore, we speculated that LL and TK might play a role in maintaining T1 sagittal angle and could be changed in the surgical procedure through osteotomy to restore the whole sagittal balance. However, few studies have been performed to explore the relationship between T1 sagittal angle and spine sagittal parameters, such as LL and TK [9,10,26]. Therefore, we conducted this retrospective study to determine the relationship between T1 sagittal angle and sagittal balance, predictors for T1 sagittal angle, and whether T1 sagittal angle could be used for osteotomy guidelines to restore sagittal balance.

It was found in our study that T1 sagittal angle was statistically correlated to SVA with moderate significance (r = 0.445, *P*<0.001), which is consistent with the finding of Knott et al [9], who reported that the value of correlation between T1 sagittal angle and SVA was 0.65 (*P*<0.001), which is larger than that reported in our study. This difference may be explained by the significant difference in sample size (119 *vs*. 52) and measurement errors between the two studies. Furthermore, we also explored the correlation between T1 tilt angle and other whole sagittal parameters (TPA and T1SPI) that were not studied in previous studies. We found that T1 sagittal angle was also correlated with TPA and T1SPI, with weak significance of 0.207 (*P* = 0.023) and 0.309 (*P* = 0.001), respectively. As T1 sagittal angle was correlated with three parameters that reflected the whole sagittal balance, we could consider T1 sagittal angle as a good predictor of overall sagittal balance, which was also reported in the study of Knott et al [9], although it has its own limits, such as not very strong significance between T1 sagittal angle and whole sagittal balance. Our correlation analysis also showed correlations between T1 sagittal angle and maxTK, maxLL, SS and PI. The significant correlation between T1 sagittal angle and maxTK and maxLL verifies our previous finding that regional spinal parameters (TK and LL) play a key role in maintaining sagittal balance, which also reflects a chain of correlation between regional sagittal parameters [10]. SS was also found to be significantly correlated with T1 sagittal angle, which can be easily understood because the geometric relationship has been verified: T1 sagittal angle = SS-(maxLL-maxTK) (maxTK was usually measured from T1 to T12, and maxLL was measured from L1 to S1). Besides, T1 sagittal angle was also found to be correlated with PI, a morphologic parameter used to define lumbar alignment [27]. Because it is generally accepted that increased PI is due to alteration of the overall anthropometric morphology and twisting mobilization of the sacro-iliac joints induced by the biomechanical conditions of sagittal disturbance [28,29]. Sagittal balance can be maintained through three main compensatory mechanisms that could occur in the spine, pelvis and/or lower limb areas, including reduction of TK/hyperextension of adjacent segments, pelvis retroversion (increase of PT and rotation of the pelvis), knee flexion and ankle extension [30,31]. Hyperextension of the adjacent segments is a common compensatory mechanism in keeping sagittal balance, for pelvis retroversion, knee flexion and ankle extension may occur secondary to hyperextension of the adjacent segments if these segments are too rigid to extend or reach their limits [30,32]. Our unadjusted and adjusted multiple regression analyses showed that TK and LL were two important predictors for T1 sagittal angle, and our unadjusted analysis showed that maxTK, maxLL and SS were primary contributors to T1 sagittal angle. However, PI was not included in the final regression equation in our study. It is widely believed that PI is described and considered unchanged in adulthood as long as the sacroiliac joints remain stable. Therefore, it might not be important contributor to the whole sagittal balance [29,33]. In the adjusted analysis, we removed SS form the equation as all the people recruited in our study were healthy volunteers without any spinal diseases and their sagittal alignment was balanced without any secondary compensatory mechanisms, such as pelvic retroversion, knee flexion and ankle extension. In addition, since pelvic parameters play a role in compensatory mechanisms [25], SS might not be a primary contributor to T1 sagittal angle and sagittal balance in healthy populations,. Therefore, our final results showed that T1 sagittal angle could be predicted by using the following regression equation: T1 sagittal angle = 0.6 * maxTK—0.2 * maxLL + 8, which further verifies the previous opinion that reduction of TK/hyperextension of the adjacent segments was an important compensatory mechanism and our previous finding that regional spinal parameters (TK and LL) played a key role in maintaining sagittal balance. Furthermore, we should also notice that maxTK plays a more important role in sagittal balance than maxLL as the coefficients of maxTK and maxLL were 0.6 and 0.2, respectively.

In the healthy population, sagittal alignment maintains balance through changing TK and LL as shown in regression equation (T1 sagittal angle = 0.6 * maxTK—0.2 * maxLL + 8), which could also provide guidelines for surgery. With increased awareness of the importance of sagittal balance in HRQOL, many surgeons pay more attention to the restoration of sagittal alignment than coronal imbalance. Therefore, our findings could provide guidelines for osteotomy because spinal surgeons could restore sagittal balance easily by changing TK and LL more easily than SS, and restore the normal relationship between TK and LL during surgery.

Although we found that TK and LL were two primary contributors to sagittal balance in healthy populations, there are some limitations in this study. First, all the subjects recruited in this study came from the outpatient clinic of our hospital, which might result in selection bias. Second, our study was a single-center study and the sample size was relatively small. Third, normative values and reliability studies for T1 sagittal balance were also neglected. Therefore, larger-scale and multicenter studies are required to gain more comprehensive insights into predictors for T1 sagittal angle in normal populations.

## Conclusion

T1 sagittal angle could be considered a useful parameter for sagittal balance in healthy populations, though it could not fully replace SVA. maxTK is the primary contributor to T1 sagittal angle, and could be predicted by using the following equation: T1 sagittal angle = 0.6 * maxTK—0.2 * maxLL + 8, whereby we could restore sagittal balance by surgically changing TK and LL. These findings may provide useful information for osteotomy.

## Supporting Information

S1 File The raw data of our article. (XLSX)

## Abbreviations

maxTK: Maximum thoracic kyphosis
maxLL: Maximum lumbar lordosis
LL: Lumbar lordosis
TK: Thoracic kyphosis
SS: Sacrum slope
PT: Pelvic tilt
PI: Pelvic incidence
SVA: Sagittal vertical axis
TPA: T1 pelvic angle
T1SPI: T1 spinopelvic inclination
HRQOL: Health-related quality of life
SPO: Smith-Petersen osteotomy
PSO: Pedicle subtraction osteotomy
VCR: Vertebral column resection

## References

[1] Schwab F, Ungar B, Blondel B, Buchowski J, Coe J, Deinlein D, et al Scoliosis Research Society-Schwab adult spinal deformity classification: a validation study. Spine 2012 5 20;37(12):1077–82. 10.1097/BRS.0b013e31823e15e2 22045006

[2] Lafage V, Schwab F, Patel A, Hawkinson N, Farcy JP. Pelvic tilt and truncal inclination: two key radiographic parameters in the setting of adults with spinal deformity. Spine 2009 8 1;34(17):E599–606. 10.1097/BRS.0b013e3181aad219 19644319

[3] La Maida GA, Zottarelli L, Mineo GV, Misaggi B. Sagittal balance in adolescent idiopathic scoliosis: radiographic study of spino-pelvic compensation after surgery. Eur Spine J. 2013 11;22 Suppl 6:S859–67. 10.1007/s00586-013-3018-8 24061971 PMC3830044

[4] Jang JS, Lee SH, Min JH, Kim SK, Han KM, Maeng DH. Surgical treatment of failed back surgery syndrome due to sagittal imbalance. Spine 2007 12 15;32(26):3081–7. 18091505 10.1097/BRS.0b013e31815cde71

[5] Lee JH, Na KH, Kim JH, Jeong HY, Chang DG. Is pelvic incidence a constant, as everyone knows? Changes of pelvic incidence in surgically corrected adult sagittal deformity. Eur Spine J. 2015 8 20. [Epub ahead of print]. 10.1007/s00586-015-4199-0 26289634

[6] Hikata T, Watanabe K, Fujita N, Iwanami A, Hosogane N, Ishii K, et al Impact of sagittal spinopelvic alignment on clinical outcomes after decompression surgery for lumbar spinal canal stenosis without coronal imbalance. J Neurosurg Spine. 2015 10;23(4):451–8. 10.3171/2015.1.SPINE14642 26140404

[7] Baghdadi YM, Larson AN, Dekutoski MB, Cui Q, Sebastian AS, Armitage BM, et al Sagittal balance and spinopelvic parameters after lateral lumbar interbody fusion for degenerative scoliosis: a case-control study. Spine 2014 2 1;39(3):E166–73. 10.1097/BRS.0000000000000073 24150436 PMC4340477

[8] Le Huec JC, Faundez A, Dominguez D, Hoffmeyer P, Aunoble S. Evidence showing the relationship between sagittal balance and clinical outcomes in surgical treatment of degenerative spinal diseases: a literature review. Int Orthop. 2015 1;39(1):87–95. 10.1007/s00264-014-2516-6 25192690

[9] Knott PT, Mardjetko SM, Techy F. The use of the T1 sagittal angle in predicting overall sagittal balance of the spine. Spine J. 2010 11;10(11):994–8. 10.1016/j.spinee.2010.08.031 20970739

[10] Pesenti S, Blondel B, Peltier E, Choufani E, Bollini G, Jouve JL. Interest of T1 parameters for sagittal alignment evaluation of adolescent idiopathic scoliosis patients. Eur Spine J. 2016 2;25(2):424–9. 10.1007/s00586-015-4244-z 26433584

[11] Qiao J, Zhu F, Xu L, Liu Z, Zhu Z, Qian B, et al T1 pelvic angle: a new predictor for postoperative sagittal balance and clinical outcomes in adult scoliosis. Spine 2014 12 1;39(25):2103–7. 10.1097/BRS.0000000000000635 25271508

[12] Yang C, Yang M, Wei X, Shao J, Chen Y, Zhao J, et al Lumbar Lordosis Minus Thoracic Kyphosis (Ll-Tk): A Novel Regional Predictor for Sagittal Balance in Elderly Populations. Spine 2016 3;41(5):399–403. 10.1097/BRS.0000000000001231 26555829

[13] Yang M, Yang C, Chen Z, Wei X, Chen Y, Zhao J, et al Lumbar Lordosis Minus Thoracic Kyphosis: Remain Constant in Adolescent Idiopathic Scoliosis Patients Before and After Correction Surgery. Spine 2016 3;41(6):E359–63. 10.1097/BRS.0000000000001258 26536436

[14] Wiedenhofer B, Furstenberg CH, Schroder K, Akbar M. Multiplan correction of a 3D deformity. Options and relevance of optimizing the thoracic kyphosis in reconstructive scoliosis surgery. Orthopade. 2011 8;40(8):672–81. 10.1007/s00132-011-1795-5 21751031

[15] Coskun Benlidayi I, Basaran S. Comparative study of lumbosacral alignment in elderly versus young adults: data on patients with low back pain. Aging Clin Exp Res. 2015 6;27(3):297–302. 10.1007/s40520-014-0274-3 25286900

[16] Yang C, Yang M, Chen Y, Wei X, Ni H, Chen Z, et al Radiographic Parameters in Adult Degenerative Scoliosis and Different Parameters Between Sagittal Balanced and Imbalanced ADS Patients. Medicine 2015 7;94(29):e1198 10.1097/MD.0000000000001198 26200633 PMC4603005

[17] Tang JA, Scheer JK, Smith JS, Deviren V, Bess S, Hart RA, et al The impact of standing regional cervical sagittal alignment on outcomes in posterior cervical fusion surgery. Neurosurgery. 2012 9;71(3):662–9; discussion 669. 22653395 10.1227/NEU.0b013e31826100c9

[18] Vialle R, Levassor N, Rillardon L, Templier A, Skalli W, Guigui P. Radiographic analysis of the sagittal alignment and balance of the spine in asymptomatic subjects. J Bone Joint Surg Am. 2005 2;87(2):260–7. 15687145 10.2106/JBJS.D.02043

[19] Protopsaltis T, Schwab F, Bronsard N, Smith JS, Klineberg E, Mundis G, et al TheT1 pelvic angle, a novel radiographic measure of global sagittal deformity, accounts for both spinal inclination and pelvic tilt and correlates with health-related quality of life. J Bone Joint Surg Am. 2014 10 1;96(19):1631–40. 10.2106/JBJS.M.01459 25274788

[20] Etemadifar M, Ebrahimzadeh A, Hadi A, Feizi M. Comparison of Scheuermann's kyphosis correction by combined anterior-posterior fusion versus posterior-only procedure. Eur Spine J. 2016 8;25(8):2580–6. 10.1007/s00586-015-4234-1 26365711

[21] Bodin A, Roussouly P. Sacral and pelvic osteotomies for correction of spinal deformities. Eur Spine J. 2015 1;24 Suppl 1:S72–82. 10.1007/s00586-014-3651-x 25501693

[22] Berjano P, Cecchinato R, Sinigaglia A, Damilano M, Ismael MF, Martini C, et al Anterior column realignment from a lateral approach for the treatment of severe sagittal imbalance: a retrospective radiographic study. Eur Spine J. 2015 4;24 Suppl 3:433–8. 10.1007/s00586-015-3930-1 25893333

[23] Zheng GQ, Song K, Zhang YG, Wang Y, Huang P, Zhang XS, et al Two-level spinal osteotomy for severe thoracolumbar kyphosis in ankylosing spondylitis. Experience with 48 patients. Spine 2014 6 1;39(13):1055–8. 10.1097/BRS.0000000000000346 24732843

[24] Akbar M, Wiedenhofer B. Sagittal deformity. Basic principles of surgical strategies. Orthopade. 2011 8;40(8):661–71. 10.1007/s00132-011-1814-6 21779881

[25] Fechtenbaum J, Etcheto A, Kolta S, Feydy A, Roux C, Briot K. Sagittal balance of the spine in patients with osteoporotic vertebral fractures. Osteoporos Int. 2016 2;27(2):559–67. 10.1007/s00198-015-3283-y 26272312

[26] Lee SH, Kim KT, Seo EM, Suk KS, Kwack YH, Son ES. The influence of thoracic inlet alignment on the craniocervical sagittal balance in asymptomatic adults. J Spinal Disord Tech. 2012 4;25(2):E41–7. 10.1097/BSD.0b013e3182396301 22037167

[27] Legaye J, Duval-Beaupere G, Hecquet J, Marty C. Pelvic incidence: a fundamental pelvic parameter for three-dimensional regulation of spinal sagittal curves. Eur Spine J. 1998;7(2):99–103. 9629932 10.1007/s005860050038 PMC3611230

[28] Upasani VV, Tis J, Bastrom T, Pawelek J, Marks M, Lonner B, et al Analysis of sagittal alignment in thoracic and thoracolumbar curves in adolescent idiopathic scoliosis: how do these two curve types differ? Spine 2007 5 20;32(12):1355–9. 17515826 10.1097/BRS.0b013e318059321d

[29] Jean L. Influence of age and sagittal balance of the spine on the value of the pelvic incidence. Eur Spine J. 2014 7;23(7):1394–9. 10.1007/s00586-014-3207-0 24509774

[30] Barrey C, Roussouly P, Perrin G, Le Huec JC. Sagittal balance disorders in severe degenerative spine. Can we identify the compensatory mechanisms? Eur Spine J. 2011 9;20 Suppl 5:626–33. 10.1007/s00586-011-1930-3 21796393 PMC3175931

[31] Yagi M, Hosogane N, Okada E, Watanabe K, Machida M, Tezuka M, et al Factors affecting the postoperative progression of thoracic kyphosis in surgically treated adult patients with lumbar degenerative scoliosis. Spine 2014 4 15;39(8):E521–8. 10.1097/BRS.0000000000000226 24480961

[32] Le Huec JC, Saddiki R, Franke J, Rigal J, Aunoble S. Equilibrium of the human body and the gravity line: the basics. Eur Spine J. 2011 9;20 Suppl 5:558–63. 10.1007/s00586-011-1939-7 21809013 PMC3175916

[33] Mac-Thiong JM, Roussouly P, Berthonnaud E, Guigui P. Age- and sex-related variations in sagittal sacropelvic morphology and balance in asymptomatic adults. Eur Spine J. 2011 9;20 Suppl 5:572–7. 10.1007/s00586-011-1923-2 21833574 PMC3175918

